# Variability in Frontotemporal Brain Structure: The Importance of Recruitment of African Americans in Neuroscience Research

**DOI:** 10.1371/journal.pone.0013642

**Published:** 2010-10-26

**Authors:** Nneka Isamah, Warachal Faison, Martha E. Payne, James MacFall, David C. Steffens, John L. Beyer, K. Ranga Krishnan, Warren D. Taylor

**Affiliations:** 1 The Duke Neuropsychiatric Imaging Research Laboratory, Duke University Medical Center, Durham, North Carolina, United States of America; 2 The Duke University School of Medicine, Duke University Medical Center, Durham, North Carolina, United States of America; 3 Department of Psychiatry, Duke University Medical Center, Durham, North Carolina, United States of America; 4 Department of Radiology, Duke University Medical Center, Durham, North Carolina, United States of America; 5 The Department of Neurosciences, Alzheimer's Research and Clinical Programs, Medical University of South Carolina (MUSC), Charleston, South Carolina, United States of America; 6 The Duke-NUS Graduate Medical School, Singapore, Singapore; Cuban Neuroscience Center, Cuba

## Abstract

**Background:**

Variation in brain structure is both genetically and environmentally influenced. The question about potential differences in brain anatomy across populations of differing race and ethnicity remains a controversial issue. There are few studies specifically examining racial or ethnic differences and also few studies that test for race-related differences in context of other neuropsychiatric research, possibly due to the underrepresentation of ethnic minorities in clinical research. It is within this context that we conducted a secondary data analysis examining volumetric MRI data from healthy participants and compared the volumes of the amygdala, hippocampus, lateral ventricles, caudate nucleus, orbitofrontal cortex (OFC) and total cerebral volume between Caucasian and African-American participants. We discuss the importance of this finding in context of neuroimaging methodology, but also the need for improved recruitment of African Americans in clinical research and its broader implications for a better understanding of the neural basis of neuropsychiatric disorders.

**Methodology/Principal Findings:**

This was a case control study in the setting of an academic medical center outpatient service. Participants consisted of 44 Caucasians and 33 ethnic minorities. The following volumetric data were obtained: amygdala, hippocampus, lateral ventricles, caudate nucleus, orbitofrontal cortex (OFC) and total cerebrum. Each participant completed a 1.5 T magnetic resonance imaging (MRI). Our primary finding in analyses of brain subregions was that when compared to Caucasians, African Americans exhibited larger left OFC volumes (F _1,68_ = 7.50, p = 0.008).

**Conclusions:**

The biological implications of our findings are unclear as we do not know what factors may be contributing to these observed differences. However, this study raises several questions that have important implications for the future of neuropsychiatric research.

## Introduction

Despite the implication from classical atlases derived from small numbers of brains, structural anatomy of the brain can vary substantially. The majority of such differences likely represent normal variations, both genetically influenced and environmentally modified, but some differences may be informative for identifying risk factors for developing neuropsychiatric disorders. Many of these differences relate to basic demographic factors, as there are differences in brain structure between the sexes [1] and established aging-related changes in brain structure [2], [3]. More recently, genetic differences have been demonstrated to be related to brain structure and function [4]. As allele frequency for many genetic polymorphisms differs based on racial and ethnic background, this leads to questions about differences in brain structure or function based on ancestry. Such potential population differences may create methodological biases if atlases or templates developed from one population were applied to a different population [5].

There is a paucity of research examining racial or ethnic differences in brain structure. Most of the evidence in the available literature is limited to neuroimaging studies which control for racial background. For example, there are differences between Caucasians and Chinese individuals in frontal, parietal, and temporal gyri morphology [5], [6], as well as in white matter anatomy [7], and the authors have speculated that some of these differences may be related to the environmental influence of the subject's native language [6]. There are also racial differences in age-related brain changes, with African-American individuals exhibiting greater aging-related increases than Caucasians in cerebral ventricle volume [8], while race is also associated with hippocampal volume in older individuals with cognitive deficits [9]. More recently, widespread differences in brain structure have been observed between Chinese and Caucasian cohorts [5], a finding particularly important for automated image processing methods which rely on population-specific brain atlases.

Although many neuroimaging studies attempt to match samples on demographic factors, they rarely test for or report differences based on racial background. One of the major reasons may be because ethnic minorities are underrepresented in clinical research, which despite policies designed to improve the inclusion of minorities in research, continues to remain problematic [10]–[20]. Although the specific reasons are unknown, there are a number of barriers to research participation that have been highlighted, such as distrust of the medical system [21], negative attitudes exhibited by “gatekeepers” including physicians, family members, community leaders [22], [23], research entry criteria [17], language and/or literacy barriers associated with the consent process or research protocols [19], [24], and lack of transportation [19]. Further, there is a body of literature that suggests ethnic minorities are not informed of research opportunities and if asked/appropriately informed, they will participate [25]. Despite this historical underrepresentation, appropriate inclusion of racial or ethnic minorities may be crucial to fully understand normal variation in brain structure and function across our broader community. This has implications for understanding the neural basis of neuropsychiatric disorders.

This study was a secondary data analysis examining volumetric MRI data gathered from healthy control subjects participating in a study of bipolar disorder. This dataset enabled us to compare volumes of temporal regions (amygdala and hippocampus), the orbitofrontal cortex (OFC), the caudate nucleus, the lateral ventricles, and total cerebral volume between Caucasian and African-American participants.

## Methods

### Ethics Statement

Subjects provided written informed consent before study procedures were performed. The study was approved by the Duke University Health System Institutional Review Board.

### Study Design

This was a secondary analysis of data collected from a study examining the pathophysiology of Bipolar Disorder. Importantly, the parent study was not designed to examine ethnic or racial differences. Participants were recruited from the community by advertisement for inclusion as healthy control subjects. Eligibility criteria included age of 18 years or older and English speaking. Although not an entry criterion, all subjects were native English speakers. Exclusion criteria included: 1) any psychiatric disorder history, including substance abuse or dependence, as detected using the National Institute of Mental Health Diagnostic Interview Schedule (Robins et al., 1981); 2) uncontrolled medical illness; 3) any current or past use of psychotropic medications; 4) pregnancy; and 5) any MRI contraindication.

### Magnetic Resonance Imaging Acquisition and Analysis

MR imaging of the brain was performed on a 1.5 T system (Signa, GE Medical Systems, Milwaukee, WI) using the standard head (volumetric) radiofrequency coil. The scanner alignment light was used to adjust head tilt and rotation so that the axial plane lights passed across the cantho-meatal line and the sagittal lights were aligned with the center of the nose. A rapid sagittal localizer scan was acquired to confirm alignment. A dual-echo fast spin-echo (FSE) acquisition was obtained in the axial plane for morphometry of cerebral structures including lateral ventricles, orbitofrontal cortex (OFC) and caudate. The FSE series had pulse sequence parameters of TR = 4000 ms, TE = 30 ms±16 kHz full imaging bandwidth, echo train length = 16, 256×256 matrix, 3-mm section thickness, 1 excitation and a 20-cm FOV. The images were acquired in two separate acquisitions with a 3-mm gap between sections for each acquisition. The second acquisition was offset by 3 mm from the first so that the resulting data set consisted of contiguous sections. An axial IR-prepped 3D series was acquired for measuring the amygdala and hippocampus, with pulse sequence parameters of TE = minimum full echo, TI = 300 ms±16 kHz bandwidth, 256×256 matrix, 1.5-mm section thickness, 1 excitation and a 24-cm FOV.

The MR images were processed at the Neuropsychiatric Imaging Research Laboratory (NIRL) by analysts blinded to subject identity and clinical data. A NIRL-modified version of MrX software was used for tissue segmentation following previously described methods [26], and was used for measures of the cerebrum, gray and white matter volumes, ventricles, and caudate. Cerebrum was measured as part of this method as a proxy for total brain volume, and included a summation of cerebral gray and white matter and CSF, but did not include the brain stem or cerebellum. As an alternative measure, we also examined the ratio of total gray and white matter to total CSF (GM+WM/CSF), although this measure was not used in models to control for total brain volume. Amygdala and hippocampus measurements were performed using the NIRL-developed software program GRID, which allows for viewing and tracing in any of three orthogonal planes, regardless of acquisition plane. Volumes were calculated by multiplying the traced area on each slice by slice thickness, and then summing volumes across slices.

Detailed measurement procedures used for the OFC [27], hippocampus [28], amygdala [29], and caudate [30] have been previously described. After training, reliability was established by repeated measurements on multiple scans before image analysts were approved to process study data. Intraclass correlation coefficients (ICCs) were: total cerebrum = 0.997, left lateral ventricle = 0.988, right lateral ventricle = 0.991, left caudate = 0.94, right caudate = 0.94, left hippocampus = 0.91, right hippocampus = 0.92, left amygdala = 0.91, right amygdala = 0.87, left OFC = 0.93, right OFC = 0.997.

### Statistical Analysis

SAS 9.1 (Cary, NC) was used for all statistical analyses. Our primary measures included both measures of each brain region and the ratio of each regional volume to total cerebral volume. Two-tailed Student *t*-tests were used to test for group differences in age and education, and the chi-square test to examine for group differences in sex representation. For primary analyses, the SAS PROC GLM procedure was used to create general linear models where regional brain volume was the dependent variable with age, race, sex, total cerebral volume and education level in years as independent variables. For analyses of regional ratios, similar models were developed without including total cerebral volume as a covariate.

## Results

The sample consisted of 77 individuals, 44 of which were Caucasian and 33 were minority representatives. Of those 33 individuals, 25 were African-American, 1 was Native American, 6 were Asian, and 1 self-identified as biracial (mixed African-American/Caucasian ancestry). For this study, we included only the Caucasian and African-American subjects. The African-American population was significantly younger than the Caucasian population but there were no significant differences in sex or education level (Table 1).

**Table 1:** Demographic characteristics.

	Caucasian N = 44	African-American N = 25	df	Test Statistic	p value
Age	46.4 (14.8)	35.6 (10.6)	67	t = 3.20	0.0021
Sex, % Female (N)	81.8% (36)	72.0% (18)	1	χ^2^ = 0.90	0.3419
Education	15.2 (1.9)	14.8 (1.7)	67	t = 0.84	0.4059

Age and education are presented in years. Standard deviation is in parenthesis.

After controlling for age, sex, and education level, the African-American population exhibited smaller total cerebral volume than Caucasians (Table 2), although there were no statistically significant differences in total gray matter, total white matter, or ventricular CSF volumes. In models examining specific brain regions, the only statistically significant difference was that African-Americans exhibited larger left OFC volumes than Caucasians. However, when regional ratios were examined (regional volume/total cerebral volume), the African-American cohort exhibited greater ratios for the right amygdala and bilaterally for the OFC (Table 2).

**Table 2:** Group differences in brain volume measures.

	Caucasian (N = 44)	African-American (N = 25)	F value	p value
Total Cerebrum	1178.3 (115.0)	1076.0 (67.9)	17.92	<0.0001
GM+WM/CSF Ratio	667.3 (81.8)	629.8 (54.8)	13.77	0.0004
Lateral Ventricles	21.6 (10.9)	15.2 (5.0)	0.04	0.8503
	0.018 (0.009)	0.014 (0.004)	1.30	0.2585
Total Gray Matter	665.2 (81.7)	627.2 (54.5)	3.13	0.0816
	0.566 (0.061)	0.583 (0.048)	0.52	0.4752
Total White Matter	444.7 (79.5)	429.6 (62.1)	2.19	0.1438
	0.377 (0.054)	0.398 (0.044)	1.79	0.1856
Amygdala, L	2.4 (0.5)	2.2 (0.5)	1.31	0.2568
	0.0021 (0.0004)	0.0021 (0.0003)	3.65	0.0607
Amygdala, R	2.4 (0.4)	2.2 (0.4)	1.96	0.1668
	0.0020 (0.0004)	0.0021 (0.0004)	6.05	0.0167
Caudate, L	4.0 (0.7)	3.8 (0.5)	1.93	0.1700
	0.0034 (0.0006)	0.0036 (0.0005)	0.19	0.6676
Caudate, R	4.4 (0.7)	4.1 (0.6)	2.64	0.1092
	0.0037 (0.0006)	0.0040 (0.0005)	0.51	0.4771
Hippocampus, L	3.6 (0.5)	3.5 (0.5)	1.34	0.2513
	0.0031 (0.0005)	0.0032 (0.0004)	0.00	0.9510
Hippocampus, R	3.5 (0.5)	3.5 (0.5)	0.01	0.9245
	0.0030 (0.0005)	0.0033 (0.0005)	1.31	0.2561
Orbitofrontal Cortex, L	6.8 (1.9)	7.8 (1.7)	7.50	0.0080
	0.0057 (0.0014)	0.0072 (0.0016)	10.79	0.0017
Orbitofrontal Cortex, R	7.6 (1.6)	8.3 (1.7)	3.38	0.0708
	0.0065 (0.001)	0.0077 (0.002)	7.10	0.0098

The top measure for each region is the mean volume (mLs) and the bottom measure is the ratio of regional volume/cerebrum (defined as GM+WM+CSF). Standard deviation is in parentheses. Each variable has 1 df, with 68 df for each model.

Due to the difference in age between the groups, in a secondary analysis we removed older Caucasian subjects from the analysis. When we removed Caucasian subjects older than 56 years, which was the oldest African-American subject, age was still significantly different between the two groups. To create a study population where age was not significantly different, we had to limit inclusion of Caucasian subjects to those age 55 years or younger, while including all African-American participants. This new study population included 31 Caucasian and 25 African-American subjects, with no significant difference in age (African-American: 35.6y, SD = 10.6y, range 20–56y; Caucasian: 39.6y, SD = 11.3y, range 20–55y; 54 df, t = 1.35, p = 0.1813), education level (African-American: 14.8y, SD = 1.7y; Caucasian: 15.3y, SD = 1.6y; 54 df, t = 1.12, p = 0.2696), or sex representation (African-American: 72.0% female, or 7/25; Caucasian: 80.6% female, or 25/31; 1 df, χ^2^ = 0.58, p = 0.4462). When we examined differences in MRI measures between these two groups, the previously observed volumetric differences persisted and the difference in left amygdala ratio was also statistically significant (Table 3).

**Table 3:** Age-matched group differences in brain volume measures.

	Caucasian (N = 31)	African-American (N = 25)	F value	p value
Total Cerebrum	1173.4 (119.4)	1076.0 (67.9)	16.60	0.0002
GM+WM/CSF Ratio	680.9 (89.5)	629.8 (54.8)	12.86	0.0008
Lateral Ventricles	20.1 (11.26)	15.2 (5.0)	0.02	0.8782
	0.017 (0.009)	0.014 (0.004)	1.07	0.3064
Total Gray Matter	678.6 (89.5)	627.2 (54.5)	2.92	0.0938
	0.580 (0.063)	0.584 (0.048)	0.48	0.4925
Total White Matter	450.7 (68.0)	429.6 (62.1)	2.19	0.1453
	0.385 (0.048)	0.398 (0.044)	1.71	0.1965
Amygdala, L	2.3 (0.5)	2.2 (0.5)	2.32	0.1341
	0.0019 (0.0004)	0.0021 (0.0004)	4.47	0.0396
Amygdala, R	2.3 (0.5)	2.2 (0.4)	1.76	0.1912
	0.0019 (0.0004)	0.0021 (0.0004)	5.50	0.0231
Caudate, L	4.1 (0.7)	3.8 (0.5)	1.94	0.1701
	0.0035 (0.0006)	0.0036 (0.0005)	0.03	0.8670
Caudate, R	4.4 (0.7)	4.1 (0.6)	2.34	0.1324
	0.0038 (0.0006)	0.0039 (0.0005)	0.29	0.5947
Hippocampus, L	3.7 (0.5)	3.5 (0.5)	0.64	0.4266
	0.0032 (0.0005)	0.0032 (0.0004)	0.01	0.9142
Hippocampus, R	3.6 (0.6)	3.5 (0.5)	0.01	0.9118
	0.0031 (0.0005)	0.0033 (0.0005)	1.23	0.2734
Orbitofrontal Cortex, L	7.0 (2.2)	7.8 (1.7)	6.06	0.0174
	0.0059 (0.0016)	0.0072 (0.0016)	7.85	0.0072
Orbitofrontal Cortex, R	7.9 (1.6)	8.3 (1.7)	2.74	0.1040
	0.0068 (0.0013)	0.0077 (0.0016)	5.61	0.0216

The top measure for each region is the mean volume (mLs) and the bottom measure is the ratio of regional volume/cerebrum (defined as GM+WM+CSF). Standard deviation is in parentheses. Each variable has 1 df, with 55 df for each model.

Finally, we tested for interactions between race and age and race and sex and their influence on these regional brain measures. These interaction terms did not achieve statistical significance in any model (data not shown).

## Discussion

Classification of individuals by race has been a long standing controversial issue in biomedical research. Consistent with the notion that “there is no biological basis for race” [31], some make the argument that race is biologically meaningless, dismissing race as a non-scientific concept [32], [33] and therefore irrelevant in research. On the other side of the argument, collecting demographic information should not be limited to age, sex or socioeconomic status, because information about race and ethnicity are useful for identifying genetic and environmental influences on psychiatric illness [34] and may have clinical utility for determining whether particular individuals in a population are more susceptible to particular diseases, more at risk for specific adverse events, or more likely to benefit from certain therapeutic interventions [35]. There is sensitivity surrounding this debate, and race and ethnicity have a history of being used “as a cause for discrimination, prejudice, marginalization and subjugation” in the United States [34]. Consequently, due to a number of factors, few studies examine racial or ethnic differences in human biology. Such research is particularly limited in the organ most associated with our identity, the brain.

Our primary finding is that when compared to Caucasians, an African-American cohort exhibited smaller cerebral volumes but larger absolute left OFC volumes. Additionally, the OFC and amygdala appear to occupy a significantly greater proportion of the total cerebral volume in the African-American cohort. Importantly, this was statistically significant in a small cohort, which suggests that small differences in racial representation across cohorts in neuroscience research may bias study results, particularly if analyses do not consider or control for racial background. Our findings are generally concordant with recent work that brain structure may vary significantly across populations of different racial or ethnic backgrounds [5].

Our two sets of analyses, where we examined either regional volumes while controlling for cerebral volume, or examining a ratio of regional volume to cerebral volume, resulted in different findings, primarily due to the differences between groups in total cerebral volume. As these different analytic techniques had different results, we included both analyses to demonstrate these differences. Interestingly, this was most apparent for the amygdala, where comparisons of amygdala volume suggested a trend for African-Americans to exhibit smaller volumes, but it appears that the amygdala in African-Americans may occupy a greater proportion of total cerebral volume. However, racial differences may influence results using either analytic technique.

Our findings have neither clear clinical implications nor clear implications for differences in brain function. The OFC and amygdala are functionally linked and contribute to stimulus assessment and face recognition. Race-related differences have previously been reported with frontal and temporal activation, including the OFC, amygdala, hippocampus and fusiform gyrus [36]–[40], however regional volume and function are not consistently linked. The difference in cerebral volume is even less clear, as studies examining sex differences in cerebral volume have suggested such “global” differences may be related to regional differences or differences in proportions of tissue types [41].

Our findings do have clear implications for neuroscience research. Racial and ethnic background accounts for some of the variability in brain structure and so this demographic needs to be consistently incorporated into neuroscience research. Although this demographic characteristic is limited and does not necessarily capture the complex genetic and environmental influences that likely underlie our findings, it cannot be ignored. This is particularly important for image processing methods dependent on population atlases [5].

Twin and family studies provide consistent evidence for the role of both genetics and environmental influences in shaping the developing brain. Heritability effects differ by brain region, with highest familial relationships in the frontal lobe and moderate relationships in the hippocampus and amygdala [42]. As one might expect from such research, genetic differences are related to brain structure and function, and these influences appear strongest for areas of the brain involved in language, attention, visual, emotional and sensorimotor processing [43]. Likewise, the frequency of many alleles, including those involved in CNS function, differs substantially across ethnic and racial populations. These two separate observations have not been explicitly united. For example, the *5HTTLPR* short/long polymorphism has been associated with differences in frontotemporal structure and function [44]–[47]. *5HTTLPR* allele frequency also varies by ancestry: African-Americans exhibit a lower frequency of the s allele (25%) than do Caucasian Americans of European descent (40–45%), while the short allele is particularly common in some Asian populations [48], [49].

Environmental factors may also influence brain structure and function. For example, socioeconomic status is related to the prefrontal cortex and hippocampus, areas responsible for executive function, language and memory [50], [51]. This effect may be mediated through numerous environmental factors, including childhood diet, access to health care, childhood adversity, or access to quality education. Importantly, such hypotheses are highly speculative, although testable.

Although it is unknown what factors contribute to these differences, our findings carry potential implications. First, these observations may help us understand clinical differences, as work examining neurobiological racial differences could augment cultural and environmental research examining how ancestral background may influence ethnic differences in the clinical presentation of mental health problems. A prime example of this is Major Depressive Disorder, which presents differently between African-American and Caucasian individuals [52]–[54], and has different risk of onset at specific life periods [55].

Second, our findings provide support for the importance of not just acquiring information about racial and ethnic background in biomedical research, but also stresses the importance of improved recruitment of ethnic minorities in clinical trials. Lack of inclusion of racial and ethnic minorities in research impacts the generalizability of trial findings and hinders subgroup analyses [56]. Inclusion of minority participants in research is critical for the generation and testing of hypotheses about which how biological, cultural, and environmental differences influence critical endpoints such as risk, treatment response, or adverse events [56]. Moreover, lack of recognition or understanding of racial differences in brain structure and function leads to the possibility of inaccurate conclusions in neuroimaging studies where racial differences are not appropriately considered.

Study limitations include self report of race. We did not acquire more detailed ancestral background which may be relevant (such as a Mediterranean versus Scandinavian background), nor did our assessment account for unrecognized racial heterogeneity in one's ancestry. Most study participants were women, which likely influences brain structure findings for sex in the analyses, although we found no interaction between race and sex on regional measures. Additionally, we did not assess other developmental factors that could moderate or mediate the relationships we observed, including personal or parental socioeconomic status, childhood exposures, trauma, or health habits. Our measure of education is an imperfect proxy for socioeconomic status, but may be important in its own right. Many of these limitations are inherent to our study design of conducting a secondary analysis of data collected through a study not designed to test for ethnic or racial differences.

Finally, our sample size of 69 individuals was small, although comparable to other neuroimaging studies examining structural differences between different racial populations [5]. We also conducted multiple comparisons between the small groups, which increases the risk of a Type I error. Had we instituted a Bonferroni correction for multiple comparisons, the alpha would change to 0.004; at this alpha, only the cerebral volume and left OFC volume would have remained significant.

These findings generate additional questions: Are there broader differences in brain structure and function across individuals of different ancestry, and what genetic and environmental factors included in the demographic assessment of race most strongly influence differences in brain structure? Do these volumetric differences contribute to heterogeneity in clinical presentation or outcomes of neuropsychiatric disease? Can comparable efforts in examining race in clinical trials provide clues about optimal pharmacotherapy choices or risk of adverse events? In order to answer these questions, we must be proactive and improve our recruitment efforts. Researchers from academia and industry must collaborate with communities to continue to explore barriers to minority recruitment into clinical research as well as discover ways to promote an understanding of the importance of research and participation by ethnic minorities.
